# Effect of alisertib, an investigational aurora a kinase inhibitor on the QTc interval in patients with advanced malignancies

**DOI:** 10.1007/s10637-017-0498-0

**Published:** 2017-08-18

**Authors:** Xiaofei Zhou, John Nemunaitis, Shubham Pant, Todd M. Bauer, Manish Patel, John Sarantopoulos, A. Craig Lockhart, Daniel Goodman, Dirk Huebner, Diane R. Mould, Karthik Venkatakrishnan

**Affiliations:** 1 Quantitative Clinical Pharmacology, Millennium Pharmaceuticals, Inc., a wholly owned subsidiary of Takeda Pharmaceutical Company Limited, Cambridge, MA USA; 2 Mary Crowley Cancer Research Centers, Dallas, TX USA; 3 Oklahoma University Medical Center, Oklahoma City, OK USA; 4 Sarah Cannon Research Institute, Sarasota, FL USA; 5 Florida Cancer Specialists, Sarasota, FL USA; 6 Institute for Drug Development, Cancer Therapy and Research Center, University of Texas Health Science Center San Antonio, San Antonio, TX USA; 7 Washington University, St. Louis, MO USA; 8 BioTelemetry, Inc, Rockville, MD USA; 9 Projections Research Inc., Phoenixville, PA USA

**Keywords:** Alisertib, QTc interval, Aurora a kinase

## Abstract

*Aims* A primary objective of this study was to investigate the effect of single and multiple doses of alisertib, an investigational Aurora A kinase inhibitor, on the QTc interval in patients with advanced malignancies. The dose regimen used was the maximum tolerated dose which was also the recommended phase 3 dose (50 mg twice daily [BID] for 7 days in 21-day cycles). *Methods* Patients received a single dose of alisertib (50 mg) on Day 1, and multiple doses of alisertib (50 mg BID) on Days 4 through to the morning of Day 10 of the first cycle of treatment. Triplicate ECGs were collected at intervals over 10 to 24 h via Holter recorders on Days −1 (baseline), 1 and 10. Changes from time-matched baseline values were calculated for various ECG parameters including QTc, heart rate, PR and QRS intervals. Alisertib pharmacokinetics were also assessed during the study, and an exposure-QTc analysis was conducted. *Results* Fifty patients were included in the QTc analysis. The upper bounds of the 95% confidence intervals for changes from time-matched baseline QTcF and QTcI values were <5 ms across all study days, time points and correction methods. Alisertib did not produce clinically relevant effects on heart rate, PR or QRS intervals. There was no evidence of a concentration-QTc effect relationship. *Conclusions* Alisertib does not cause QTc prolongation and can be concluded to not have any clinically relevant effects on cardiac repolarization or ECG parameters at the single agent maximum tolerated dose of 50 mg BID.

## Introduction

Alisertib is a selective small molecule inhibitor of Aurora A kinase that is being developed for the treatment of advanced malignancies. Aurora A belongs to a highly conserved family of serine/threonine protein kinases that also includes Aurora B and Aurora *C. aurora* A and Aurora B are expressed in all actively dividing cells, while Aurora C expression is largely restricted to dividing germ cells [1]. Aurora A localizes to centrosomes and the proximal mitotic spindle during mitosis where it functions in a diverse set of mitotic processes. In addition, Aurora kinases may be active in oncogenic signaling pathways, and these diverse roles remain the subject of laboratory and clinical studies [2].

Evidence to support Aurora A kinase as a therapeutic target for the treatment of malignancies comes from several sources. First, the Aurora A kinase gene is amplified or overexpressed, or both, in many tumors including colon, breast, pancreatic, and bladder cancers, as well as certain lymphomas, leukemias, and myeloma [3–7]. In epithelial ovarian cancer (EOC), Aurora A kinase has been reported to be frequently upregulated and associated with worse clinical outcome. Some evidence indicates that dysregulation of Aurora A kinase may be an early event in EOC with a key role in tumor progression [8]. Aurora A overexpression in human cancers has been correlated with increased aneuploidy and centrosome amplification [9]. The overexpression of Aurora A kinase results in the transformation of normal cells, supporting the hypothesis that Aurora A is an oncogene [3]. Lastly, in a number of different experimental systems, Aurora A inhibition leads to mitotic delays and severe chromosome congression and segregation defects, followed by cell death [10–13].

Preclinically, alisertib exhibited minimal activity against human ether-à-go-go related gene (hERG) current (IC_50_ and K_i_ > 100 μM) and hence was not anticipated to cause prolongations in the QTc interval of the electrocardiogram (ECG) in humans. However, formal assessment of this potential is an important consideration in drug development, as QTc interval prolongation is associated with an increased risk of cardiac arrhythmias, particularly torsades de points (TdP), an arrhythmia which may spontaneously lead to ventricular fibrillation and sudden death [14, 15]. Accordingly, a formal assessment of the effect of single and multiple doses of alisertib on the QTc interval was undertaken. Alisertib is a cytotoxic agent and as it cannot be administered to healthy subjects, this study was conducted in patients with advanced solid tumors or lymphomas. Accordingly, the study did not include a placebo-control or a positive-control (such as moxifloxacin, which is known to prolong QTc interval), which is consistent with typical approaches used in the evaluation of the effects of anticancer agents on QTc [16, 17]. The study was conducted using the maximum tolerated dose of 50 mg twice daily (BID) alisertib, which also represented the upper end of the clinical dose range in phase 2/3 development.

## Methods

### Study design

This study was an open-label, phase 1 study in patients with advanced solid tumors or lymphomas. A primary objective of the study was to evaluate the effect of single and multiple oral doses of alisertib on the QTc interval. This objective was investigated in the first cycle of treatment. Another objective was to evaluate the effect of esomeprazole and rifampin on the pharmacokinetics of alisertib. The methods and results of this drug-drug interaction analysis will be published separately, and hence only details pertinent to the QTc assessment are provided here. The safety data from this study will also be reported with the drug interaction data.

Patients were screened up to 28 days prior to the first dose of alisertib to assess eligibility. Eligible patients were then enrolled into the study and received a single dose of 50 mg alisertib on Day 1 of Cycle 1 followed by 50 mg BID alisertib on Day 4 through until the morning dose on Day 10 of Cycle 1. Patients attended the study center on the day prior to the first dose (Day −1) for baseline assessments and returned on each of Days 1 to 4 and 10 of Cycle 1 for study assessments. Alisertib was administered in the study center on Days 1, and 10, and was administered at home by the patients on Days 4 to 9. The clock time of dosing on Day 10 coincided with that of the 0 h time point on Days −1 and 1. On Days −1, 1 and 10, patients were fasted (no food or drink except water) from 2 h prior to the 0 h time point until completion of the 4-h assessments. On other days the patients were asked to fast from 2 h before until 1 h after dosing. Upon completion of Cycle 1 (24-days), patients could continue in subsequent cycles which are not reported here.

The study was conducted in accordance with the International Conference on Harmonization guideline for Good Clinical Practice and the ethical principles of the Declaration of Helsinki. The study was conducted in 6 centres in the United States and was approved by the institutional review board(s) and/or local independent ethics committee(s) at each center. The study was registered on ClinicalTrials.gov (NCT01844583). The study was conducted between June 2013 and August 2014.

### Patients

Eligible patients were male or female with histologically or cytologically confirmed metastatic and/or advanced solid tumors or lymphomas for which standard curative or life-prolonging treatment did not exist or was no longer effective or tolerable. Patients were aged 18 years or older, had an Eastern Cooperative Oncology Group performance status of 0 or 1, had an expected survival longer than 3 months from enrolment, and had adequate hematologic, renal and hepatic function. All patients had to provide written informed consent and comply with contraceptive requirements. Key exclusion criteria included treatment with any anticancer therapy or investigational agent within 4 weeks prior to Day 1, recurrent nausea and/or vomiting within 14 days prior to Day 1 or any known gastrointestinal abnormality or procedures that could interfere with or modify absorption or tolerance to alisertib. Patients were excluded if they required treatment with clinically significant enzyme inducers within 14 days prior to Day 1 or during the study, had a medical condition requiring use of pancreatic enzymes, or daily, chronic or regular use of proton pump inhibitors or histamine (H2) receptor antagonists, were taking QT-prolonging drugs with a risk of causing torsades de pointes (TdP). Patients with a history of myocardial infarction, unstable symptomatic ischemic heart disease, any cardiac arrhythmia (except sinus arrhythmia), thromboembolic events, or other cardiac condition within 6 months of Day 1 were also excluded, as were patients with a history of risk factors for TdP, family history of long QT syndrome, or Brugada syndrome. Patients were excluded if they had an abnormal 12-lead ECG at screening indicating a second- or third-degree atrioventricular block or intermittent block, or with QRS >110 ms, QTcF >480 ms, PR interval > 200 ms or any arrhythmia considered by the investigator to be clinically significant or had sustained blood pressure > 160 mmHg or <90 mmHg (systolic), BP >100 mmHg or <65 mmHg (diastolic) or resting heart rate < 50 beats per minute (bpm) or >100 bpm at screening or predose.

### Pharmacokinetic assessments

Blood samples for analysis of alisertib and its metabolites M1 (alisertib acylglucuronide) and M2 (*O*-desmethyl alisertib) were collected at intervals from 0 (predose) to 72 h after the single dose of alisertib on Day 1, and from 0 to 10 h on Day 10.

Plasma samples were analyzed for alisertib and its metabolite concentrations using validated liquid chromatography tandem mass spectrometry (LC-MS/MS) methods utilizing solid phase extraction procedure. The quantitation range for the alisertib assay was 5 to 2500 ng/mL, the assay precision, expressed as percent coefficients of variation (%CV) for quality control (QC) samples ranged from 4.0 to 7.1% and the mean accuracy, expressed as percent bias, for QC samples ranged from −0.8 to 2.0%. The quantitation range for the M1 assay was 2.00 to 1000 ng/mL, the assay precision ranged from 3.0 to 4.1% and the mean accuracy ranged from −1.5 to 0.3%. The quantitation range for the M2 assay was 2.00 to 1000 ng/mL, the assay precision ranged from 3.0 to 4.3% and the mean accuracy ranged from −1.1 to 0.7%.

Pharmacokinetic parameters were estimated using non-compartmental analysis with Phoenix™ WinNonlin® Version 6.2 (Pharsight Corporation, Mountain View, CA, USA). The following pharmacokinetic parameters were calculated on Days 1 and 10: maximum observed plasma concentration (C_max_), and first time to C_max_ (T_max_). Area under the concentration time curve from time 0 to the last quantifiable time point (AUC_0-last_), area under the concentration-time curve from time 0 extrapolated to infinite time (AUC_0-inf_), and terminal half-life (t_1/2_) were calculated on Day 1 only, and area under the concentration time curve from time 0 to 10 h postdose (AUC_0-10h_), and accumulation ratio (R_ac_) were calculated on Day 10 only. The AUC parameters were estimated using the linear-log trapezoidal rule.

### QTc assessments

Continuous 12-lead digital ECGs were obtained using a Holter ECG recorder on Days −1, 1 and 10. Three Holter ECGs (approximately 1 min apart) were extracted on each day at times that matched the times of Day 1 postdose PK sampling (up to 24 h on Days −1 and 1, and 10 h on Day 10). The Day −1 triplicate ECGs served a time-matched baseline data.

### Statistical analysis

The ECGs were read centrally and all QTc data represented the means of the 3 replicates at each time point. Two correction methods were used for all analyses of QTc: individual patient correction (QTcI = QT/RR^b^) and Fredericia’s correction (QTcF = QT/RR^(1/3)^). For QTcI, all pairs of QT and RR interval data collected on Day −1 including the Day 1 predose measurement were analyzed by linear regression to define a slope (b) for each patient, which was then used to calculate the individual correction for that patient. Heart rate (HR), PR interval, QRS duration and ECG morphologies were also analyzed.

The ECG data set comprised all available ECGs extracted from the Holter monitor from all patients who were dosed on Day 1. Only patients who received at least 4 days of dosing prior to Day 10 (ie, all protocol-specified doses on Days 6 to 10) were used for the analyses of the effect of alisertib on Day 10 ECG parameters (as this duration of dosing ensures achievement of >90% of steady-state exposures on Day 10).

The change from the time-matched Day −1 baseline in QTcI was the primary endpoint. This change was calculated by subtracting the Day −1 mean QTcI from the time-matched Day 1 or Day 10 mean QTcI. QTcF was the secondary endpoint. In addition to analyses of central tendency, categorical analyses for each QTc interval were also conducted for Days 1 and 10 including absolute QTc >450, >480 or >500 ms, and change from baseline in QTc of >30 or >60 ms, QRS duration >110 ms and 25% increase from baseline, and PR interval > 200 ms and 25% increase from baseline.

The primary analysis was a repeated measures mixed effects linear model and all inferences were based on least squares means. For each time point, a 1-sided 95% upper confidence bound on the mean change from baseline was presented. All statistical analyses were conducted using SAS version 9.2 (SAS Institute, NC, USA). A sample size of 36 patients was expected to provide a 1-sided 95% upper confidence bound with a width less than 2.5 ms, assuming a standard deviation of QTcI change from baseline of approximately 9 ms. Approximately 45 patients were planned to be enrolled in order to obtain a total of 36 evaluable patients.

### PK-QTc analysis

The relationships between plasma alisertib concentrations and corresponding change from time-matched baseline QTcI, QTcF and HR were analyzed using non-linear mixed effects modelling (NONMEM version 7.2, Icon Development Solutions, Dublin, Ireland). The final models were used to simulate the predicted size of the effect of alisertib on changes of QTc from baseline.

## Results

### Patient disposition and demographics

A total of 55 patients were enrolled and received at least 1 dose of alisertib. All enrolled patients were evaluable on Day 1 (ie, completed the Holter monitoring on Days −1 and 1) and 41 (75%) patients were evaluable on Day 10 (ie, completed Holter monitoring on Days −1 and 1, and also received all prescribed doses for Days 6 to 10). The median age of the patients was 61 years (range 32 to 80 years) and the majority of patients were women (64%), White (87%) and not Hispanic or Latino (84%). Fifty four patients had advanced solid tumors and one patient had mantle cell lymphoma.

### Pharmacokinetics

**Fig. 1:**
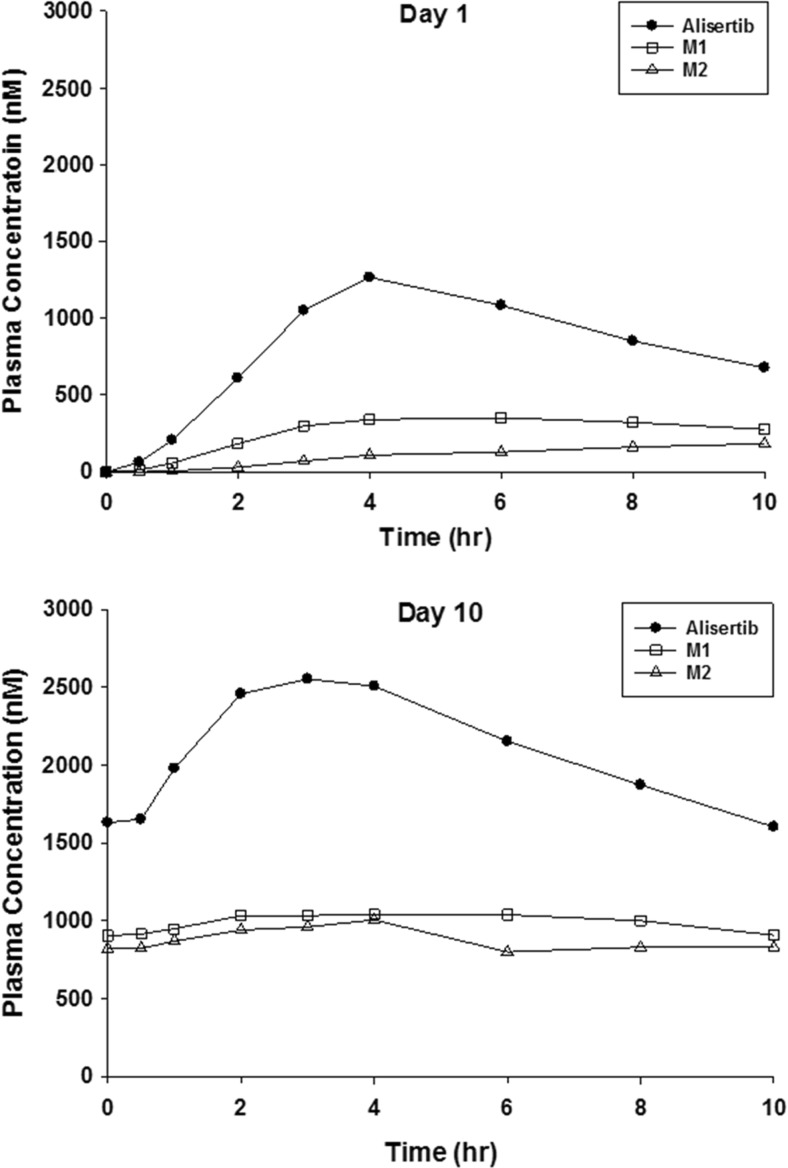
Plasma concentration versus time profiles for alisertib and its metabolites following single (Day 1) and multiple dosing (Day 10)

Concentration-time profiles of alisertib and its metablites M1 and M2 following single and multiple dosing administration of alisertib are shown in Fig. 1. Following single and multiple doses of alisertib, median T_max_ for alisertib was achieved at 4 and 3 h, respectively (Table 1). The mean t_1/2_ of alisertib following single-dose alisertib was approximately 16 h. Median T_max_ and mean t_1/2_ for the metabolite M1 were similar to alisertib. The median T_max_ for metabolite M2 was achieved at 10 and 4 h, following single and multiple doses of alisertib, respectively and the mean t_1/2_ of M2 was approximately 22 h. The mean ratios of AUC_0-last_ for M1 and M2 compared to alisertib following a single dose of alisertib were 0.45 and 0.41, respectively and the mean ratios of AUC_0-10h_ for M1 and M2 compared to alisertib following multiple doses of alisertib were 0.44 and 0.42, respectively. The mean ratios of C_max_ for M1 and M2 compared to alisertib were 0.30 and 0.13, respectively on Day 1 and 0.40 and 0.37, respectively on Day 10.

**Table 1:** Summary of pharmacokinetic parameters

Analyte	Day	N	Pharmacokinetic Parameters^a^						
			C_max_ (nM)	T_max_ (h)	AUC_0-last_ (nM.h)	AUC_0-10h_ (nM.h)	AUC_0-inf_ (nM.h)	t_1/2_ (h)	R_ac_
Alisertib	1	55	1512 (41)	4 (1, 23)	18,845 (39)^b^	N/A	18,313 (42)^c^	16.4 (6.8)^c^	N/A
	10	39	2760 (35)	3 (0, 8)	N/A	20,085 (38)	N/A	N/A	3.5 (3.3)
M1	1	55	353 (80)	4 (2, 23)	5775 (123)^b^	N/A	4556 (104)^d^	16.4 (6.7)^d^	N/A
	10	39	676 (179)	3 (0, 8)	N/A	5304 (201)	N/A	N/A	N/A
M2	1	55	174 (71)	10 (4, 72)	6885 (78)^b^	N/A	5740 (122)^e^	22 (5)^e^	N/A
	10	39	943 (50)	4 (1, 10)	N/A	7778 (52)	N/A	N/A	N/A

N/A not applicable

^a^Values are geometric means (% coefficient of variation) for C_max_ and AUC parameters, median (minimum, maximum) for T_max_, and arithmetic mean (standard deviation) for t_1/2_ and R_ac_

^b^ *N* = 53

^c^ *N* = 35

^d^ *N* = 34

^e^ *N* = 15

### Effect of alisertib on ECG parameters

**Fig. 2:**
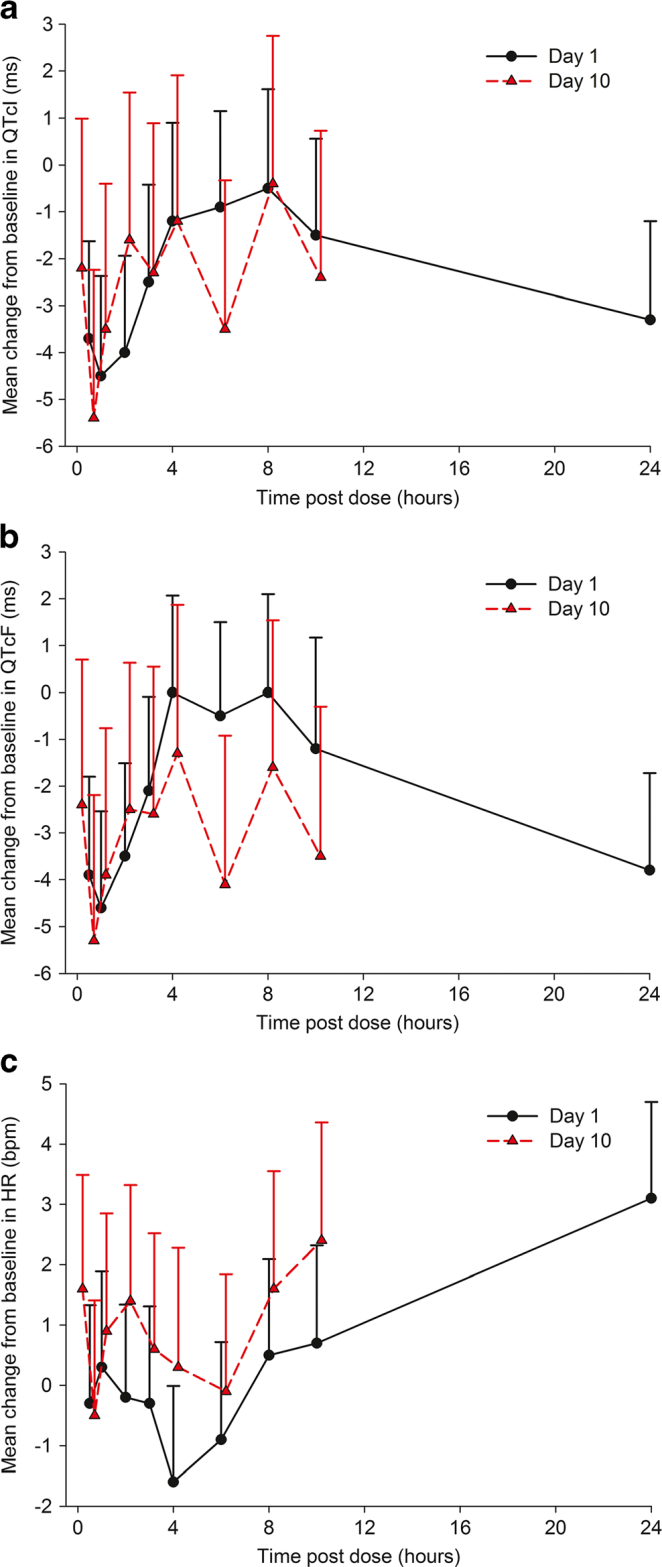
Mean changes from time-matched baseline in QTcI (**a**), QTcF (**b**) and heart rate (**c**)

The mean changes from time-matched baseline values and their 95% upper confidence bounds for QTcI, QTcF and heart rate are shown in Table 2 and Fig. 2. For QTcI the mean values ranged from −5.4 msec (Day 10 at 0.5 h) to −0.4 msec (Day 10 at 8 h). The maximum 1-sided 95% upper confidence bound was 2.75 msec (Day 10 at 8 h). There were no appreciable differences between Day 1 and Day 10 values of changes from time-matched baseline QTcI. As the maximum upper confidence bound of the change of QTcI from baseline was within the expected variation for this parameter, and no trends for values of change by study day or time point were noted, it can be concluded that alisertib did not cause prolongation of repolarization on the ECG.

**Table 2:** Mean changes from time-matched baseline in QTcI, QTcF and Heart Rate and their 95% upper confidence bounds

Time postdose (hours)	QTcI (ms)				QTcF (ms)				Heart Rate (bpm)			
	Day 1		Day 10		Day 1		Day 10		Day 1		Day 10	
	Mean	95% UCB	Mean	95% UCB	Mean	95% UCB	Mean	95% UCB	Mean	95% UCB	Mean	95% UCB
0	N/A	N/A	−2.2	0.99	N/A	N/A	−2.4	0.7	N/A	N/A	1.6	3.49
0.5	−3.7	−1.63	−5.4	−2.24	−3.9	−1.8	−5.3	−2.19	−0.3	1.33	−0.5	1.41
1	−4.5	−2.37	−3.5	−0.4	−4.6	−2.54	−3.9	−0.76	0.3	1.89	0.9	2.85
2	−4	−1.94	−1.6	1.54	−3.5	−1.51	−2.5	0.63	−0.2	1.34	1.4	3.32
3	−2.5	−0.42	−2.3	0.89	−2.1	−0.09	−2.6	0.55	−0.3	1.31	0.6	2.52
4	−1.2	0.9	−1.2	1.91	0	2.07	−1.3	1.87	−1.6	−0.01	0.3	2.28
6	−0.9	1.15	−3.5	−0.33	−0.5	1.5	−4.1	−0.92	−0.9	0.72	−0.1	1.84
8	−0.5	1.61	−0.4	2.75	0	2.1	−1.6	1.54	0.5	2.09	1.6	3.55
10	−1.5	0.56	−2.4	0.73	−1.2	0.9	−3.5	−0.3	0.7	2.32	2.4	4.36
24	−3.3	−1.2	N/A	N/A	−3.8	−1.72	N/A	N/A	3.1	4.70	N/A	N/A

N/A not applicable

The maximum QTcF change was −5.3 msec (Day 10 at 0.5 h), and there was no change at 2 time points (Day 1 at 4 and 8 h). The maximum 1-sided 95% upper confidence bound was 2.10 msec (Day 1 at 8 h). The findings for change of QTcF from baseline confirmed the findings of the primary endpoint, QTcI.

The maximum 1-sided 95% upper confidence bound for heart rate was 4.70 bpm (Day 1 at 24 h). Day 10 values were slightly higher than those from Day 1, but the differences were in the range of 1 bpm at most time points. No clear trend was noted for the time course of the observations.

Mean changes in PR interval from time-matched baseline values were minimal, ranging from −2.0 to 2.7 ms. No consistent trends were noted for any study day or time point. Mean changes in QRS duration from time-matched baseline values were also minimal, ranging from −0.3 to 2.0 ms.

QTcI values >500 ms were observed in one patient on Day −1 and on Day 1, but no patients had QTcI >500 ms on Day 10 or QTcF values >500 ms on any day. No patients had changes from baseline in any of the QTc intervals of >60 ms. Likewise, no patients had PR intervals >200 ms and >25% increased from baseline, or QRS intervals of >110 ms and >25% increased from baseline.

There were no emergent diagnostic findings indicative of repolarization changes or other important effects on the heart based on assessment of ECG morphology.

### Exposure-QTc analysis

**Fig. 3:**
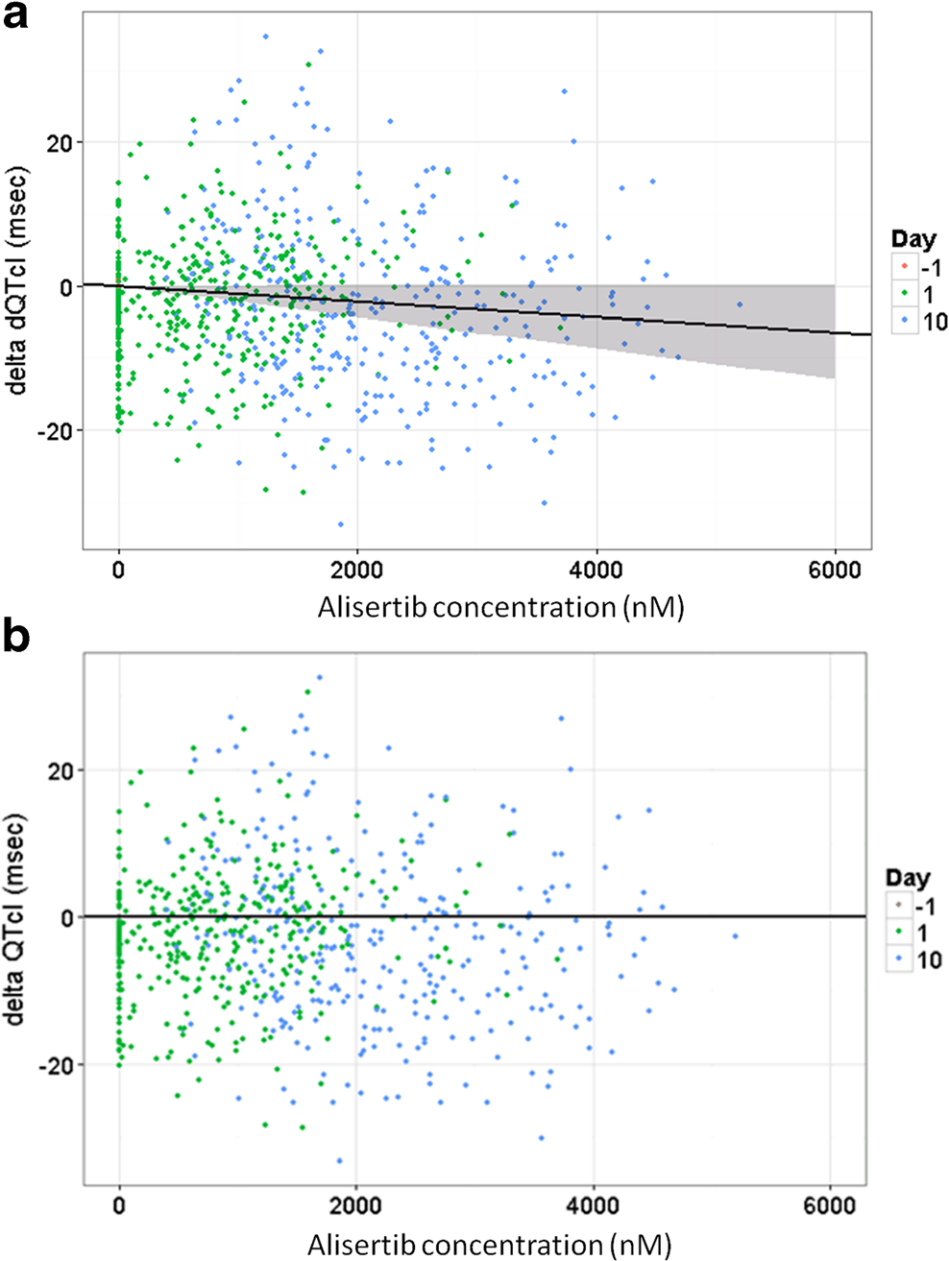
dQTcI Models – Bootstrap results for linear (**a**) and no-effect models (**b**). Symbols are observed data, with symbol color delineating study day. The black line is the line for the median of the bootstrap (1000 datasets) parameters, with the grey ribbon showing the 90% CIs for the line. CI = confidence interval, dQTcI = delta individually corrected QT interval

Four classes of structural models were examined to describe the relationship between alisertib plasma concentration and corresponding change from time-matched baseline in QTcI (dQTcI) and HR: baseline (no drug effect) models, linear models, E_max_ models and sigmoid E_max_ models. Even when a model with a linear effect of concentration was evaluated, the estimated slope (msec/nM) of the concentration-dQTcI relationships was negative (dQTcI: −0.000952 with a 95% CI of −0.00305 to 0.00115), supporting the lack of any readily apparent alisertib concentration-related QT prolongation. The effect of alisertib concentration on dQTcI was best described by a “no effect” model indicating no detectable concentration-effect relationship. The rationale for the use of the “no effect” model as the final model also provided the most conservative estimate of effect on QTc in simulation analyses. The percent standard error (SE%) and the between subject variability (BSV) for the intercept in the “no effect” model were 10.1 ms, and 7.17 ms, respectively. This model assumed dQTcI had a natural between-subject variability about a population value of 0 that was unaffected by alisertib concentration. Covariate analysis concluded that the sex, body mass index (BMI), and ECOG status of the patients did not influence the dQTcI. The bootstrap analysis is shown in Fig. 3.

The change in QTcF (dQTcF) from baseline was investigated as a secondary endpoint, and the results were generally similar to those found for the primary endpoint, dQTcI (data not shown).

Alisertib concentration did not appear to influence HR. The model assumed HR had a natural between-subject variability about a population value (75 bpm) that was unaffected by alisertib concentration.

The distribution of the change in QTc from baseline (dQTcI and dQTcF) for a simulated population of 1000 patients at the geometric mean of the maximum concentration at steady-state for a 50 mg BID dose regimen (2.76 μM) is summarized in Table 3. For 1000 patients simulated from the final models, the percentage of dQTc values above the threshold of 30 ms at the nominal C_max_ was 0.2% and 0.1% for QTcI and QTcF, respectively. For 1000 patients simulated from the final models, the percentage of dQTc values above the upper threshold for concern (>60 ms) at C_max_ was 0% for both QTcI and QTcF.

**Table 3:** Summary of simulated effect size at C_max_

Metric	Median	Mean	SD	CV%	Minimum	5th percentile	95th percentile	Maximum
dQTcI	−0.062	0.325	9.68	2980	−27.5	−15.2	16.7	32.0
dQTcF	−0.071	0.323	9.39	2911	−26.6	−14.8	16.1	31.0

*SD* Standard deviation, *CV* Coefficient of variation

## Discussion

The effect of 50 mg BID dosing of alisertib on the QTc interval was evaluated in 55 patients with cancer. There was no clinically relevant effect of single- and multiple-dose administration of alisertib at a dose of 50 mg BID on the ECG QT interval.

The upper bounds of the 95% CIs for changes from time-matched baseline values of QTc were <5 msec across study days (Days 1, 10), time points, and QTc correction methods (QTcI and QTcF). While absolute estimates of effects on QTc were slightly different across the various QT correction methods, analyses of data using all 3 correction methods evaluated consistently supported the conclusion of no cardiac repolarization effects. Alisertib did not produce clinically relevant effects on HR or on PR or QRS intervals, and there were no emergent diagnostic findings indicative of repolarization changes or other important effects on the heart based on the assessment of ECG morphology. The calculated geometric mean steady-state unbound C_max_ of alisertib on Cycle 1, Day 10 was 0.069 μM in this study (based on a total C_max_ of 2.76 μM and an alisertib in vitro free fraction of 0.025) and the observed lack of effect of alisertib on QTc is consistent with nonclinical findings, in which alisertib exhibited minimal activity against human ether-à-go-go related gene current (IC_50_ and K_i_ > 100 μM).

Population-based models were used to examine the relationships between HR, dQTcI and dQTcF, and alisertib concentrations. No alisertib concentration-effect relationships were discernible from these analyses, consistent with findings from the statistical analyses in supporting a lack of effect on the QT interval.

A previous Aurora kinase A inhibitor, VX-680/MK-0457, was withdrawn from development due to apparent QTc prolongation [18, 19]. The results from the current study concluded that QT prolongation is not a class effect of AAK inhibitors, and support the use of standard cardiac safety monitoring in the clinical development of alisertib.
